# Mode of Effective Connectivity within a Putative Neural Network Differentiates Moral Cognitions Related to Care and Justice Ethics

**DOI:** 10.1371/journal.pone.0014730

**Published:** 2011-02-25

**Authors:** Ricardo Cáceda, G. Andrew James, Timothy D. Ely, John Snarey, Clinton D. Kilts

**Affiliations:** 1 Department of Psychiatry and Behavioral Sciences, Emory University School of Medicine, Atlanta, Georgia, United States of America; 2 Brain Imaging Research Center, Psychiatric Research Institute, University of Arkansas for Medical Sciences, Little Rock, Arkansas, United States of America; 3 Candler School of Theology, Emory University, Atlanta, Georgia, United States of America; University College London, United Kingdom

## Abstract

**Background:**

Moral sensitivity refers to the interpretive awareness of moral conflict and can be justice or care oriented. Justice ethics is associated primarily with human rights and the application of moral rules, whereas care ethics is related to human needs and a situational approach involving social emotions. Among the core brain regions involved in moral issue processing are: medial prefrontal cortex, anterior (ACC) and posterior (PCC) cingulate cortex, posterior superior temporal sulcus (pSTS), insula and amygdala. This study sought to inform the long standing debate of whether care and justice moral ethics represent one or two different forms of cognition.

**Methodology/Principal Findings:**

Model-free and model-based connectivity analysis were used to identify functional neural networks underlying care and justice ethics for a moral sensitivity task. In addition to modest differences in patterns of associated neural activity, distinct modes of functional and effective connectivity were observed for moral sensitivity for care and justice issues that were modulated by individual variation in moral ability.

**Conclusions/Significance:**

These results support a neurobiological differentiation between care and justice ethics and suggest that human moral behavior reflects the outcome of integrating opposing rule-based, self-other perspectives, and emotional responses.

## Introduction

A coherent system of morals, including social norms and normative ethics, holds human psyches and societies together. Traditionally, psychological theories of moral behavior were based on a rational approach that was an outgrowth of Kantian philosophy, with Lawrence Kohlberg as its major proponent [1]. Later, neo-Kohlbergian theorists advanced theories emphasizing a context-dependent process involving components of moral decision making in addition to reasoning [2]. On the other hand, there is a long standing position supporting the notion that moral behavior is an emotion-based process [3]. These theorists further proposed that moral conflicts engage the automatic, implicit recruitment of cognitive processes supporting early components of moral decision making [4]. Such processes are referred to as moral sensitivity, a precondition to moral judgment, or the innate detection and initial interpretive processing of a moral issue that may be triggered by a potential moral conflict. Human functional neuroimaging studies of responses to moral dilemmas have led to considerable advances in understanding the neural representation of moral behavior. A variety of experimental paradigms, ranging from passive picture viewing [5], [6] to active decision making [2], [7], [8] , have been used to impose moral dilemmas in neuroimaging settings, and a consistent core of activated brain regions is associated with moral issue processing: dorsolateral prefrontal cortex (DLPFC), medial prefrontal cortex (mPFC), orbitofrontal cortex, anterior (ACC) and posterior cingulate cortex (PCC), precuneus, anterior temporal cortex, superior temporal sulcus (STS), ventral striatum, insula, and amygdala. Furthermore, activity of regions within this basic brain network has been shown to be modulated by the different qualities of a moral dilemma, including agency [9], [10], emotion, [11], [12] and intentionality [13], [14], [15].

Moral sensitivity can be considered justice or care oriented on the basis of the properties of the moral stimulus and the ethical stance of the individual. Justice issues and ethics are associated with human rights and the application of universal ethical principles in the form of rules, obligations, and codes, whereas care issues and ethics are associated with human situational needs and the application of social emotions, such as empathy and altruism [1]. Robertson et al. (2007) demonstrated that sensitivity to moral issues is similarly associated with the activation of three primary brain regions: the mPFC, dorsal PCC, and posterior STS (pSTS) [16]. Functional neuroimaging studies of subjects making moral judgments have demonstrated distinct patterns of brain activation in situations in which moral outcomes are rational versus emotionally favorable, findings consistent with a care-justice typology of moral behavior [2], [17].

Traditionally, the application of functional magnetic resonance imaging (fMRI) to the identification of the neural correlates of behavior has been based on defining the magnitude and extent of the blood-oxygenation-level–dependent (BOLD) response for a behavior of interest. In a prior report [16], we used this neuroactivation analysis approach to inform moral theory by attempting a functional segregation of neuroactivations related to moral reasoning about the ethics of justice from those related to reasoning about the ethics of care. We found only partial support for differing patterns of neuroactivations associated with moral sensitivity to issues of justice and to issues of care. In this report, we readdress this comparison by analyzing functional *integration* within a putative network of brain areas involved in this moral cognition. Recent developments in functional neuroimaging design and analysis have allowed investigators to further distinguish cognitive processes on the basis of their patterns of neural network activation. Briefly, functional connectivity analyses identify brain areas where activity temporally correlates with a ‘seed region’ and thus a functional neuroanatomic model can be generated [18]. Additionally, effective connectivity analyses, such as Structural Equation Modeling (SEM), that examine the direct influence of one brain region on the activity of another brain region characterize the strength and directionality of functional interactions within a predefined anatomical model of involved brain regions [19]. To obtain a deeper understanding of the determinants of moral sensitivity, we expanded on our previous study [16] by using connectivity analysis to test the hypothesis that care and justice ethics have different neural substrates.

## Results

### Neuroactivation analysis

An SPM5 neuroactivation analysis served as a localization analysis for care and justice issue processing and replicated previous findings of care issue- and justice issue–related BOLD responses. Consistent with the putative moral cognition network, the care>neutral and justice>neutral contrasts exhibited common brain regional activations in the left pSTS, PCC/precuneus, and mPFC (Table 1). However, a voxel-wise care>justice issue contrast indicated differential activation of the precuneus and right DLPFC for care issue sensitivity, whereas the justice>issue care contrast revealed greater activation of the left DLPFC, insula, inferior parietal lobule, STS, precuneus, and precentral gyrus for justice issue sensitivity (Table 1).

**Table 1:** Anatomical and stereotaxic locations of neural activations related to the implicit recognition of care or justice moral issues (p<0.005, k≥5).

Contrast	Region (Brodmann area)	Voxel T	Cluster size		Talairach coordinates	
				x	y	z
*Care>neutral*						
	Precuneus (7)	6.04	255	8	−60	32
	Insula (L)	5.22	7	−30	−22	20
	Posterior cingulate cortex (31)	4.92	14	0	−51	28
	Posterior STS (L 22)	3.79	7	−59	−43	10
	Inferior parietal lobule (L 40)	3.59	7	−56	−42	28
	Medial frontal cortex, polar (9)	3.55	12	3	51	23
*Justice>neutral*						
	Posterior cingulate cortex (31)	5.75	405	3	−51	28
	Precuneus (L 7)	5.09	14	−15	−71	44
	Posterior STS (L 22)	5.56	293	−62	−51	21
	Inferior parietal lobule (L 7)	4.87	−	−48	−45	28
	Posterior STS (R 22)	3.47	9	53	−48	21
	Insula (R)	3.27	9	48	−40	20
	Medial frontal gyrus, polar (R 9)	3.23	5	3	51	23
*Care>justice*						
	Precuneus (31)	4.35	36	−3	−68	18
	DLPFC (R 8)	4.06	17	39	31	43
*Justice>care*						
	Posterior STS (L 22)	5.69	148	−45	−40	17
	Inferior parietal lobule (L40)	5.27	7	−45	−33	45
	Insula (L13)	5.21	8	−36	−25	20
	Precuneus	4.64	35	−15	−53	40
	Precentral gyrus (R 6)	4.35	34	42	−4	30
	DLPFC (L 6)	3.47	6	−33	8	47

### Functional connectivity

An initial psychophysiological interaction (PPI) analysis used an unconstrained model-free approach to compare the mode of functional connectivity for the processing of care or justice moral issues with seed regions confined to the three major brain regions comprising the putative moral cognitive neural network. Iterative PPI analyses demonstrated different patterns of functional connectivity related to moral sensitivity for care (care>neutral) and justice (justice>neutral) issues (Table 2). For the frontal pole (FP seed), justice issues were associated with positive correlation of the FP temporal waveform with activity in the dorsal ACC, right ventromedial prefrontal cortex, inferior parietal cortex, and left precentral gyrus; negative connectivity was observed for the left dorsal ACC and thalamus. Care issues exhibited positive FP connectivity with the dorsomedial prefrontal cortex and bilateral insula; negative connectivity was observed for the PCC. For the left pSTS seed, justice issues were associated with positive connectivity with the pregenual (rostral) anterior cingulate cortex (rACC), medial (polar) prefrontal cortex (PFC), right STS, and left precuneus; negative connectivity was observed for the dorsal striatum, anterior PCC, and left dorsal premotor cortex. Care issues exhibited positive left pSTS connectivity with the left thalamus, right middle temporal gyrus, and left precentral and postcentral gyri; negative connectivity was observed for the precuneus, ventral ACC, and precuneus. For the PCC seed, justice issues were associated with positive connectivity with the rACC and dorsal ACC, left ventral precentral gyrus, and right inferior frontal cortex (pars opercularis); negative connectivity was observed for the ventral ACC and ventral striatum. Care issues exhibited positive PCC connectivity with the left inferior frontal cortex (pars orbitalis) and left pre-supplementary motor area (pre-SMA); negative connectivity was observed for the medial (polar) PFC and left precentral gyrus. These brain-wide functional connectivity analyses identified the rACC as a region exhibiting task-related connectivity (p_uncorrected_<0.005, k>5) with two or more seed regions, particularly for justice issue processing, and was thus entered as a component of the path model for effective connectivity analysis.

**Table 2:** Patterns of functional connectivity for the frontal pole, posterior cingulate cortex, and left superior temporal sulcus seeds related to moral sensitivity for care and justice issues (p<0.005, k≥5).

Region (Brodmann area)	t-score	Cluster size	Talairach coord.			Region (Brodmann area)	t-score	Cluster size	Talairach coord.			Region (Brodmann area)	t-score	Cluster size	Talairach coord.		
			x	y	z				x	y	z				x	y	z
**Seed Region: Frontal Pole (3, 51, 23 mm)**																	
*Increased connectivity*																	
dmPFC (8)	3.95	21	−3	40	42	vmPFC (R 10)	4.08	9	21	59	12						
Insula (R)	3.83	13	45	3	0	dACC (R 24)	4.39	6	6	10	33						
Insula (L)	3.78	8	−33	−6	−3	Precentral gyrus (L4/6)	3.97	12	−36	−5	19						
						Inferior parietal cortex (R 40)	4.21	10	59	−51	21						
*Decreased connectivity*																	
PCC (31)	3.85	32	3	−51	28	dACC (L 32)	4.12	7	−24	14	40	Precuneus	4.88	26	21	−38	46
												(R 7)					
						Thalamus (L)	3.18	6	−12	−17	16	Inferior parietal lobule (R 40)	4.31	16	42	−44	50
												Precuneus (7)	3.5	25	3	−47	43
**Seed region: Posterior Cingulate Cortex (0, −51, 28 mm)**																	
*Increased connectivity*																	
Inferior frontal gyrus (L 47)	4.07	18	−48	23	−11	rACC (L 32)	3.78	7	−6	41	9	Pre-SMA ( 6)	5.05	30	−6	8	51
Pre-SMA (L 6)	5	8	−12	17	47	Precentral gyrus (L6)	3.87	9	−48	−5	11	Superior frontal gyrus (R 8)	4.68	29	9	43	42
						Inferior frontal cortex (R44)	3.97	6	50	12	10	Inferior frontal gyrus (R 44)	3.88	5	50	32	−2
						dACC (R 32)	4.57	6	9	22	32	Inferior parietal lobule (L 40)	4.05	18	−27	−35	53
												Insula	3.77	5	27	−40	24
*Decreased connectivity*																	
Frontal pole (9)	4.74	37	3	54	28	vACC (R 24)	3.97	19	9	26	−1	vmPFC (L 11)	5.35	9	−6	29	−12
Precentral gyrus (R 4)	4.71	7	39	−12	45	Ventral striatum	3.92	43	−12	26	−5	vlPFC (R 47)	4.31	23	45	17	−11
												dACC (32)	5.03	34	−3	11	36
												Middle frontal gyrus (R 9)	3.62	10	50	5	40
**Seed Region: Left Posterior Superior Temporal Sulcus (−59 −43 10 mm)**																	
*Increased connectivity*																	
Thalamus (L)	3.8	7	−18	−17	5	rACC (L32)	3.73	9	−3	47	9	vmPFC (L 11)	4.19	7	12	26	−8
Middle temporal gyrus (R 39)	3.43	6	48	−78	22	Medial prefrontal cortex (L10)	3.69	6	−15	62	8	Thalamus (L)	4.34	9	−3	−6	0
Precentral gyrus (L6)	4.68	10	−45	−7	37	Superior temporal sulcus (R22)	3.44	5	59	−34	20	SMA (6)	4.22	9	12	−11	63
Postcentral gyrus (L40)	3.68	12	−53	−32	53	Precuneus (L19)	3.68	22	−33	−83	34	Postcentral gyrus (L 3)	3.65	20	−18	−31	72
*Decreased connectivity*																	
dACC (R 24)	4.14	8	15	29	2	Caudate nucleus	4.4	7	9	18	6	Frontal pole	3.64	10	6	45	27
dACC (L 24)	4.09	19	−15	29	2	PCC (L 31)	4.31	54	−6	−39	28	vlPFC (L 47)	3.5	6	−39	31	−15
Inferior parietal lobule (L 40)	4.51	10	33	−44	54	Precentral gyrus (L 6)	3.55	9	−53	−1	41	Precuneus (7)	3.83	6	0	−62	40
Precuneus (L 7)	6.62	27	6	−65	36												

Abbreviations: PFC, prefrontal cortex; dm, dorsomedial; vl, ventrolateral; vm, ventromedial; r, rostral; v, ventral; L, left; R, right.

Additional PPI analyses localized voxels for which the BOLD response was temporally congruent with that of the seed ROI for the direct contrast of care and justice issue processing (Table 2). For the FP seed, justice issues were associated with greater connectivity with the dorsal precuneus and right inferior parietal cortex; no voxel clusters exceeded the statistical thresholds indicating greater connectivity for care versus justice issues for the FP seed. For the left pSTS seed, care issues were associated with greater connectivity with the ventromedial PFC, thalamus, SMA, and left postcentral gyrus and anterior STS, while justice issues exhibited greater left pSTS connectivity with the frontal pole, left ventrolateral PFC, and precuneus. For the PCC seed, care issues were associated with greater connectivity with the pre-SMA, dorsomedial frontal cortex, right inferior frontal cortex, and left inferior parietal lobule, while justice issues exhibited greater PCC connectivity with ventromedial and ventrolateral PFC, dorsal ACC, and right DLPFC.

### Effective connectivity

Exploratory SEM established the interregional covariances of activity to define their directionality and strength for connectivity models generated from the four regions of interest (ROI) (FP, PCC, pSTS, and rACC) comprising the constrained anatomical model that optimally fit data from each of the three conditions (neutral, care, and justice). The individual path coefficient, standard deviation and t score of the significant (p<0.05 versus null) pathways for the three conditions are listed in Table 3. Table 4 lists the fit indicators for the best performing models obtained from SEM. Each model significantly fit the observed covariance structure for the condition data from which it was generated, but SEM could not converge to a stable solution when fit to another condition's data. In other words, each model only fit the data of the condition used to generate it and no other condition's data.

**Table 3:** Structural equation modeling connectivity coefficients for neutral, care, and justice conditions.

*Neutral*				
	PCC	FP	STS	ACC
PCC	-	0.205	0.943	-
		(0.021)	(0.023)	
		9.85	41.5	
FP	-	-	−0.062	1.04
			(0.001)	(0.001)
			−91	1690
STS	-	0.913	-	-
		(0.001)		
		5390		
ACC	-	-	1.11	-
			(0.001)	
			2150	
Care				
	PCC	FP	STS	ACC
PCC	-	0.185	0.965	-
		(0.029)	(0.031)	
		6.49	31	
FP	-	-	1.09	-
			(0.001)	
			1830	
STS	-	0.918	-	-
		(0.001)		
		1526.04		
ACC	0.299	0.885	−0.205	-
	(0.023)	(0.013)	(0.026)	
	13	69.8	−7.99	
*Justice*				
	PCC	FP	STS	ACC
PCC	-	-	0.945	0.201
			(0.001)	(0.001)
			875	205
FP	-	-	1.08	-
			(0.001)	
			2504	
STS	1.36	3.72	-	-4.18
	(0.002)	(0.002)		(0.002)
	570	1490		−2160
ACC	-	-	1.1	-
			(0.001)	
			1890	

Values show significant path coefficients, (standard deviation), and t-value versus null.

See text for definitions of brain region abbreviations.

**Table 4:** Fit parameters of best performing models obtained form SEM for neutral, care, and justice conditions.

		Neutral model	Care model	Justice model
Neutral data				
	min t	9.851	-	-
	rmsea	0.582	-	-
	AIC	872.061	-	-
	rmr	0	-	-
	GFI	0.805	-	-
	AGFI	0.513	-	-
	PGFI	0.322	-	-
Care data				
	min t	-	6.491	-
	rmsea	-	0.575	-
	AIC	-	357	-
	rmr	-	0	-
	GFI	-	1	-
	AGFI	-	1	-
	PGFI	-	0.3	-
Justice data				
	min t	-	-	569.9
	rmsea	-	-	0.575
	AIC	-	-	333
	rmr	-	-	0
	GFI	-	-	1
	AGFI	-	-	1
	PGFI	-	-	0.3

- Lisrel could not converge upon a stable solution.

min t = minimum T score; rmsea = root mean square error of approximation.

AIC =  Akaike Information Criterion; rmr = standardized root mean residual.

GFI = goodness of fit; AGFI = adjusted GFI; PGFI = parsimonious GFI.

**Figure 1:**
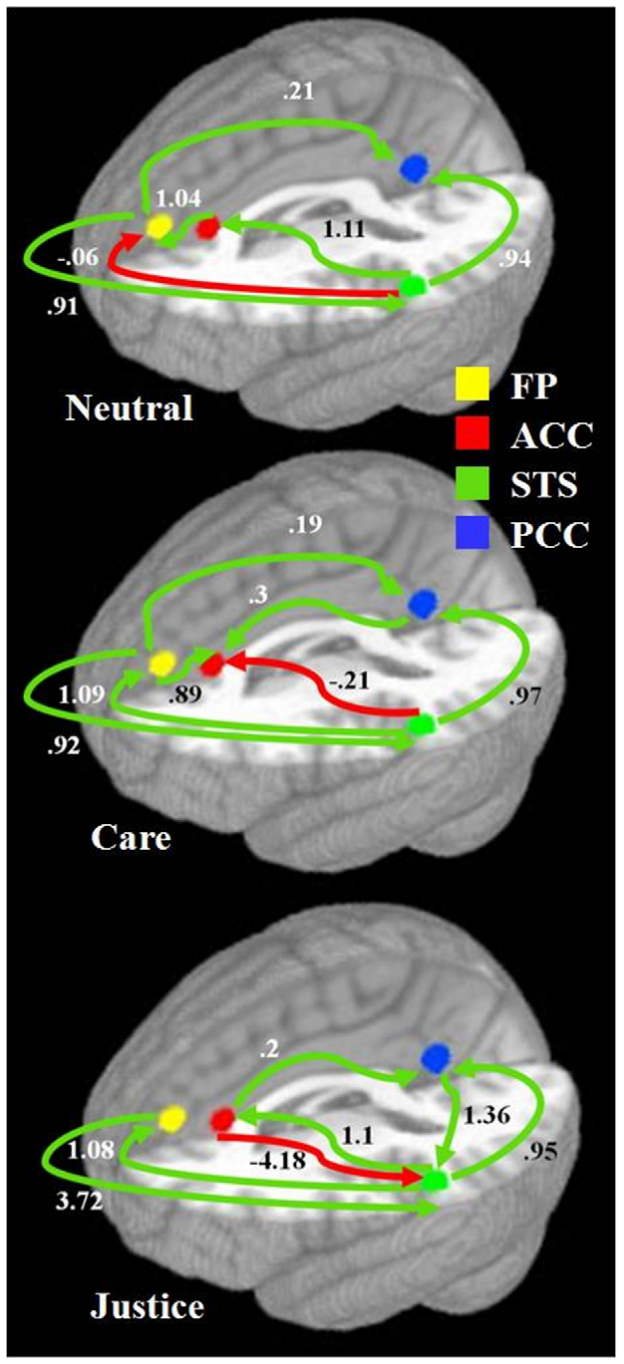
Model of brain regions involved in moral cognition: the frontal pole (yellow), rostral anterior cingulate cortex (red), left superior temporal sulcus (green), and precuneus/ posterior cingulate cortex (blue). Effective connectivity within a hypothetical neural network involved in the implicit recognition of moral issues (FP: frontal pole; rACC: rostral anterior cingulate cortex; STS: left posterior superior temporal sulcus; PCC: precuneus/posterior cingulate cortex). Circles highlight significant path coefficients.

We compared the issue conditions for each pathway to identify those pathways that are preferentially used to support the processing of care versus justice issue dilemmas. This analysis tested for differences in connection direction and strength between care and justice moral cognitions and thus compared each paths contribution to the path model between the moral issue conditions. Whether the moral issue reflected a justice or care ethic modulated the route and strength of neural information processing within the putative neural network (Figure 1). All pathwise comparisons t-statistics are reported with Bonferroni correction for the number of comparison made (n = 19). For justice issue processing, greater connectivity coefficients for the FP→pSTS path (β = 3.72 vs β = 0.92, t = 65.9, p<0.0026) and pSTS→rACC path (β = 1.1 vs β = −0.21, t = −2.6, p<0.0026) were observed relative to care issues. Moreover, a significant negative path coefficient for the rACC→pSTS path (β = −4.18) was observed for justice issues, while significant connectivity for this path was not observed for care issues (Table 3). Conversely, greater effective connectivity was observed for the FP→rACC (β = 0.89) and FP->PCC (β = 0.19) paths for care versus justice issues as these paths did not exhibit significant connectivity for justice issues (Table 3).

Compared to neutral issues, both care (β = 1.09 vs β = −0.06, t = 42.8, p<0.0026) and justice (β = 1.09 vs β = −0.06, t = 42.5, p<0.0026) issues were associated with significant increased coupling for the pSTS→FP pathway. Compared to the processing of neutral issues, the implicit processing of care moral issues was associated with a shift from a positive to a negative modulatory influence of the pSTS on the rACC (β = −0.21 vs β = 1.11, t = 2.64, p<0.0026), a reversal of the coupling between the rACC and FP, and a shift from an uncoupled to a positively coupled (β = 0.3) relationship between the PCC and rACC (Figure 1). Compared to the processing of neutral issues, justice moral issues were associated with a robust negative coupling of the rACC→pSTS pathway (β = −4.18), greater positive connectivity for the FP→pSTS pathway (β = 3.72 vs β = 0.91, t = 66.1, p<0.0026), a reciprocated influence of the PCC on the pSTS (β = 1.36), loss of influence of the rACC on the FP, and a reversal of the direction of significant path connectivity between the rACC and PCC (Figure 1).

### Relationship between moral judgment ability and brain activity for justice issue sensitivity

**Figure 2:**
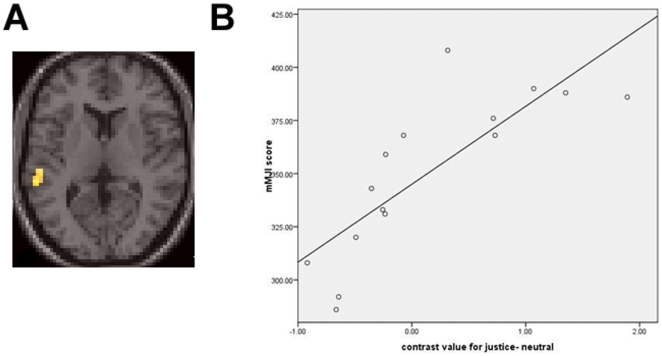
Participants' modified Moral Judgment Interview (mMJI) scores showed positive correlation with the extent of left posterior superior temporal sulcus (pSTS) activation in the justice-neutral contrast (A), and illustrated in B for a spherical volume (5 mm radius) centered on. −54, −39, 8 mm; r2 = 0.648, p<0.0001. k = >5).

Individual variation in the modified Moral Judgment Interview (mMJI) scores correlated positively with BOLD responses in the supplementary motor area (SMA) and left STS (Figure 2) for the justice>neutral contrast; BOLD responses in the right dACC were negatively correlated with individual mMJI scores (Table S1). Individual variation in the BOLD response for the justice>neutral contrast accounted for 64.8% of the variance in the mMJI scores (F−test, p<0.001). We additionally explored this behavior-neural processing association by comparing “low” and “high” subgroups derived by splitting the sample based on the median mMJI score of 359 (equivalent to the transition from Stage 3 to Stage 4 in Kohlberg's model of moral development). The mMJI scores differed significantly between the split samples (316±22 versus 380±16; p<0.0001). For justice issues, the subgroup of subjects with higher mMJI scores exhibited greater activation in the left STS, pre-SMA and SMA, and lesser activation of the dACC, and the visual, temporal, ventral prefrontal, and motor cortex than the subgroup of subjects with lower mMJI scale scores (Table S1).

For the effective connectivity analysis, stepwise regression analysis indicated that path coefficients for the pSTS→PCC path best predicted moral ability defined by the mMJI scores, explaining 59.8% of the variance [F(1,15), p<0.04]. The second step added the rACC→PCC path. Together, these two paths (pSTS→PCC and rACC→PCC) explained 82.4% of the variance in MJI scores (p<0.005). The constrained anatomical model for justice issue sensitivity contained 7 paths (Figure 1); therefore, these two PCC inputs significantly predicted mMJI scores for a Bonferroni corrected statistical threshold (p = 0.007). Both coefficients had a negative beta weight, that is, the mMJI scores increased as these path coefficients decreased.

## Discussion

Because moral judgments of care versus justice issues are related to differing facts, suppositions, and perspectives, it seems reasonable to expect that justice and care moral cognitions would be related to differing modes of neural information processing. In addition to a partial functional segregation of justice and care issues by neuroactivation analysis, we observed that care and justice issues are differentiated by the modes of functional and effective connectivity within pathways which comprise a putative neural network for moral cognition. Neural activation analysis revealed that the magnitude of DLPFC, inferior parietal cortex, STS, insula and precuneus activation differentiated care and justice cognitions. A model-free analysis of functional connectivity suggested that the interpretive detection of care and justice moral issues was associated with differing patterns of functional coupling for brain areas (FP, left pSTS, PCC) comprising this putative neural network for moral cognitions. A model-based analysis of effective connectivity indicated that moral sensitivity for care and justice issues was associated with distinct directions and strengths of information processing between the FP, left pSTS, PCC, and rACC. Furthermore, individual variation in moral development modulated the patterns of neuroactivation and of interregional connectivity related to moral sensitivity for justice issues.

### A functional neuroanatomical typology of moral sensitivity

In our prior neuroactivation study [16], we interpreted the results as evidence that moral sensitivity is a form of social cognition and self-referential thinking in which the FP is engaged to provide access to knowledge of self, the pSTS as a means of social perspective taking, and the PCC as a means of autobiographical memory recall in the service of interpretive awareness of moral conflicts. We adhere to these functional inferences in interpreting the interactions between these brain areas related to the present connectivity analyses of the neural basis of care and justice ethics. Additionally, the interpretive framework for the roles of the rACC in moral sensitivity and its proposed neural network focuses on its role in cognitive control processes related to error awareness [20] and conflict processing [21], perhaps particularly for emotional conflict [22], [23]. Other functional interpretations for the connectivity analysis results are, however, possible. In addition to their consistent implication in the processing of moral reasoning and emotion [24], [25], the cortical midline structures of the putative network are also associated with the ‘default-mode network’ [26], [27] autobiographical memory [27], [28], mentalizing or the theory of mind [29] and the mirror neuron system [30].

The pSTS emerged as a central node in a neural information processing model that differentiates care and justice ethics. Care and justice ethics theoretically differ in the importance of social perspectives [31] so it is not surprising that information processing for an area such as the STS, which is critical to social perception [32], [33], would be differentially engaged by these two types of moral issues. The large STS region comprises the superior and middle temporal gyri along its banks, and, posteriorly, the angular gyrus in its transition into the inferior parietal lobe. The STS parses rapidly changing and complex multimodal sensory information and extracts social meaning from it (for review [32], [33]). Within social cognition, the STS region is associated with the attribution of mental states to others referred to as theory of mind or mentalizing [34], [35], [36], and the perception and expression of social emotions such as trustworthiness [12], [13], [37], cooperation [38], altruism, [39], agency [14] and empathy [40], [41]. The complex social cognitive functions of the STS are supported by its reciprocal connections with visual, auditory, and somatosensory cortex, as well as higher order prefrontal and parietal association cortex [42], [43]. Functional subdivisions of the STS stress an anterior-posterior gradient of specialization, although the myriad functions of the STS are thought to reflect the ability of this brain region to use task-dependent network connections to support different cognitive operations [44]. Consistent with this notion, network connectivity analyses support a typology of moral sensitivity based on neural information flow to and from the pSTS.

Compared to the neural processing of neutral issues, care issue processing reflects a flow of pSTS information to the FP and a shift of the modulatory influence of the pSTS on rACC activity from a positive to negative one. A parsimonious explanation, based on prior functional attributions to the pSTS, is that the interpretive detection of care issues involves the biasing of FP processes of self-projection, while inhibiting the affective representations of the rACC, by the pSTS-related processes of social perspective-taking and social emotions such as altruism [39] or empathy [40], [41]. Social mentalizing would thus appear to drive the interpretation of care issues. Such network interactions are consistent with the view that care ethics are related to social contexts, inferences, and emotions [31]. The further association of care issues with PCC input to the rACC suggests that autobiographical memory recall, self awareness [45], [46], personal beliefs [47] and positive social emotions [6] may bias the affective properties of this type of moral conflict. By this pathway, the interpretive awareness of a current care moral issue could be informed by memories of past moral situations, decisions, and outcomes.

In contrast to care issues, moral sensitivity for justice issues was characterized by a convergence of information from the rACC, PCC, and FP to the pSTS. A striking strengthening of the positive influence of the FP on left pSTS activity was observed when justice issues were detected rather than care issues, with the loss of FP inputs to the other regions comprising the putative moral cognition network. Functions attributed to the FP include the representation of self-knowledge, other person knowledge, and mentalizing or theory of mind processes [48]. Within moral cognition, FP activation has been associated with emotionally intense personal dilemmas [2], [17], a finding consistent with its role in evaluative and self-referential judgment [49]. In reciprocating its pSTS inputs, the FP may bias the social situational perspective provided by the left pSTS by imposing a self-perspective for justice issue processing. In this way, the neural representation of care and justice ethics differ. The detection of justice moral issues was also associated with a robust negative connectivity coefficient for the rACC→pSTS pathway in which increased activity in the rACC predicted decreased activity in the left pSTS. The engagement of the error detection and/or affective processing functions of the rACC appear to negatively influence pSTS responses to justice issues. In other words, the perception of a violation of putative ethical principles, resulting in unfair treatment, inhibits social perspective taking related to justice issue awareness. Finally, justice ethics was associated with a reciprocated influence of the PCC on left pSTS activity, which suggests that PCC functions related to self awareness [45], [46], personal beliefs [47] positive social emotions [6] and recall of autobiographical memories of ethical violations modulates the predictive social perceptions of the pSTS. It is noteworthy that in early stages of Alzheimer's disease pronounced deficits in metabolic activity in the PCC [50] occur in the apparent absence of significant alterations of moral behavior [51]. These findings suggest that the exact contributions of the functional subdivisions of the precuneus/PCC [52] to moral cognitions need to be further elucidated.

The preferential role of the ACC for justice versus care ethics is supported by the results of the model-free functional connectivity analyses in which ACC co-activation was observed for the FP, PCC, and pSTS seeds for justice issues, but not for care issues. Notably, the dorsal ACC responses to justice issues were negatively correlated with moral development or ability, and were greater in individuals with lower rather than higher mMJI scores. Perhaps moral ability depends on a form of repetition suppression of ACC responses to violations of justice ethics so that rule violations are detected with increasing automaticity.

It is widely recognized that individuals differ markedly in their moral development or ability [53]. In a recent fMRI study [54], the right DLPFC response to violations of social norms by others scaled inversely with individual variation in the consistency of moral orientation to arguments related to case moral dilemmas. A preliminary determination of the neural basis of such variability focused on the correlation between BOLD contrast values for moral issue processing and mMJI scores, and a split sample comparison of BOLD contrast values for subject subgroups that exhibited higher and lower ability to reason about moral issues, based on their MJI scores. For justice issue processing, greater estimated moral ability was associated with greater responses in the SMA, pre-SMA, and left STS, and lesser responses in the dorsal ACC. Association with an SMA/pre-SMA mechanism implicated in the switching from habitual to controlled action selection [55], [56] suggests that higher moral development demands both the inhibition of immoral action tendencies and the facilitation of moral actions. Within the putative moral cognition network, higher moral ability for justice issues was also associated with a diminishing influence of the pSTS and rACC on PCC activity, suggesting that moral development is reflected in the level of biasing of PCC functions such that higher ability is related to more independent functioning of the PCC. A functional inference is that an autobiographical perspective that is less modulated by social and performance perspectives enables the development of justice ethics.

### Relevance of a disassociated neural representation of justice versus care ethics to moral theory

Like moral psychology, other fields of study of normative judgment have undergone similar parcellation. Moral philosophy has also been divided between two camps. Utilitarians, epitomized by John Stuart Mill, defend the pursuit of ‘the greater good’ and, as adherents to consequentialism, judge the moral worth of an action by its results in effecting the greatest amount of good for the greatest number of people. In contrast, deontologists such as Immanuel Kant propose universal moral principles that should be observed despite ‘the greater good’ and determine the moral worth of an action by examining its inherent value (for review [57]). A study of normative judgments in the law has shown that juries' decisions are not entirely dispassionate and rationally-based but can be strongly influenced by emotion [58]. In economics, emotion has also been found to interfere with the rational motivation of obtaining maximal profit predicted by neoclassical economics and game theory [59]. In all of these fields, human behavior does not follow rigidly one of the two extremes but usually transits along a middle fluctuating path.

Our results support the notion that complex human behavior reflects the integration of opposing forces in the forms of cognitive rule-based responses and social-emotional based responses [17], [54], and points to the notion that humans, rather than having either a rational or an intuitive mind, are biased toward a given moral cognitive outcome by the interactions within the corresponding neural network at a given point in time. It is most likely that this network should interact with other regions mediating executive (i.e., DLPFC, posterior parietal cortex) and emotional processes (i.e., amygdala, insula) in order to yield a given moral cognitive outcome. Although brain network responses can predict behavioral outcomes [60], real-life determinants of human behavior are still to be found. We have identified variation in moral development as a predictor of specific neural activations related to moral issues. Other trait and state factors (e.g.,education, training/profession, mood, early or recent stressful events, or physiological states) that influence human moral decision making have yet to be characterized as to their neural correlates. In addition to the inherent lure of understanding normal human decision making, results of this line of research may have a profound impact on the study of the pathophysiology of mental illness and immoral behavior.

### Limitations

The design of this moral sensitivity study and methods of estimating functional neural connectivity are associated with assumptions and experimental limitations that temper the meaningful conclusions to be drawn. In using connectivity analyses to parse the neural representations of care and justice ethics, we assumed that the covariance of changes in activity between brain regions reflects their organization into functional neural networks. We also sought to characterize the influence of individual variation in moral development or ability on the neural processing of moral dilemmas using scores provided by a mMJI instrument. The MJI represents a long-standing instrument for assessment of moral judgment ability that is linked to dominant moral stage theory [61]. Here we attempted to enhance its content validity by specific application to the justice issue scenarios used as moral dilemmas in the fMRI study. In the prior study [16], we went to great lengths to produce ecologically relevant moral stimuli and matched non-moral stimuli and also to select a professionally homogeneous sample to minimize the effects of different training histories. However, it is important to keep in mind that our sample was composed of specialized subjects with extensive work experience and training in analytical and problem solving skills in business settings and our findings might thus not be generalizable to the general population. Additionally, this study of moral sensitivity conformed to an implicit processing task in which subjects were asked to pay attention and signal any “important” issues, but without explicit decision making or judgments. The number of button pressings was higher for the justice than care conditions. This has been ascribed to the higher affinity of business professionals for conflicts of a justice ethics [16]. Our sample also consisted only of male subjects. In addition to anecdotal observations regarding sex differences in moral intuition, sex differences in neuroactivations related to moral sensitivity have also been recently reported [62]. Future functional connectivity studies should further address potential difference between genders within moral cognition networks, as well as possible variations across the menstrual cycle, as has been observed in other complex behavioral responses [63]. The functional interpretation of the network level interactions that underlie moral sensitivity and differentiate information processing for care and justice moral issues is based on selective functional attributes for the component brain regions. Each of these regions contributes to many implicated functions, and other brain regions (*e.g*., amygdale, right temporoparietal junction [9], [68]) have been implicated in moral conflict processing, yet were not considered here. Indeed, the validity of this practice of reverse inference has been criticized [64]. Therefore, these functional interpretations should be considered as preliminary inferences. Finally, the major thrust of this study was to assess and compare the effects of care and justice sensitivity on the strength and directionality of neural information processing within a putative neural processing network for moral cognitions, and the relationship of these functional variables to individual variation in moral ability. The supporting data analyses were conservatively corrected for the effects of multiple comparisons to control possible Type I error. The brain-wide neuroactivation and functional connectivity analyses furnished preliminary evidence that care and justice sensitivity were the product of separable neural neural processing. However, these analyses were intended as anatomical localization approaches for specifying the anatomical model for SEM-based path analysis with results reported by uncorrected statistical thresholds to balance Type I and Type II error rates.

### Summary

The interpretive detection of a moral issue recruits a network of brain regions that supports self-projection and that differs markedly in mode of network functional connectivity for moral violations that reflect ethical compromises of a care or justice typology. While replicating a finding of modest differences in neuroactivation magnitude [16], distinct modes of functional and effective connectivity differentiate the neural processes related to care and justice ethics. Care reasoning was characterized by a functional equilibration between pSTS (empathic response) and FP (self-knowledge) information processing. Yet justice reasoning was characterized by pSTS activity that was driven by FP (self-perspective) and ACC (rule violation/rule-based learning/emotional regulation) responses. Within the studied circuit, the FP may exert executive control over STS function and thus modify the empathic output of the circuit, shifting to a moral outlook driven by rules and self-interest. Similar to the previous findings of increasing numbers of functional neuroimaging studies [65], we found that the neural organization of human behavior is better defined by the mode of functional connectivity between involved brain regions than by the magnitude of neural responses in the component regions. These results support the neurobiological differentiation of care and justice issues and suggest that human moral behavior is the outcome of integrating rule-based, self-other perspectives, and emotional responses.

## Materials and Methods

### Ethics statement

Following thorough discussion of the intent and risks associated with the study, subjects gave written informed consent to participate in a study protocol approved by the Institutional Review Board of Emory University.

The fMRI data for this study were obtained from a neuroactivation study of moral sensitivity for care and justice dilemmas [16]. Study details related to the subject characteristics, stimuli and tasks, fMRI acquisition, and analyses are provided in the original study description [16]. Salient details are provided here. Participants in this study were male adult business professionals engaged in the implicit recognition of ecologically relevant moral dilemmas. Subjects had no personal history of psychiatric or neurologic disorders by self-report and were financially compensated for their participation. Subjects were pre-tested for reading speed using the Sight Word Efficiency subtest of the Test of Word Reading Efficiency [66]. The mean estimated standard score for the subject pool was 94.3±7.1, indicating average sight word efficiency for the group. The study was conducted in the Biomedical Imaging Technology Center at Emory Hospital.

Using a familiar business case scenario, based on the workday context of a fictional character, “Bob,” five types of issues emphasizing moral (justice or care) or non-moral (neutral, strategic or tactical) content were presented within evolving story segments. We developed a total of 41 story segments, 12 contained moral issues (describing six justice and six care issues), 12 contained nonmoral issues (describing six strategic and six tactical issues), and 17 contained neutral events. Each story segment was 2–3 sentences (and 23–35 words) in length and was written at approximately the ninth grade level of reading comprehension, according to the Flesch–Kincaid scale that assesses readability based on the average number of syllables per word and the average number of words per sentence. The 41 story segments were presented visually one at a time for 15 s each without interruptions between them. Story segments were presented in a block design in which two segments of a given type were separated by a neutral story segment, e.g., two care issues, followed by a neutral story segment, followed by two strategic issues, etc. The analysis of fMRI responses focused on the justice, care, and neutral issues. Justice issues and ethics rely on principles of fairness and impartiality and emphasize the liberating of others from injustice. Care issues and ethics rely on benevolence and compassion in responding to others' contextually embedded need with the goal of liberating them from their state of need. Neutral story segments similarly described the workday context but involved general events that did not pose a justice or care moral issue. All of the story segments went through a two-step content validation process by expert raters.

Neuroactivation and connectivity analyses used comparisons to neutral scenarios to control for activations related to reading and comprehension, context processing, issue salience, task attentional and sensorimotor demands, and the maintenance of task rules. To identify the implicit processing of moral issues and nonmoral events, subjects were asked to respond (with an MRI-compatible button box) when they identified an “important point or issue” in the story segments. Functional MRI acquisitions (Siemens 3T Tim-upgraded Trio MRI scanner) generated BOLD contrast images using T2-weighted gradient echo, echo-planar pulse sequences. Image processing involved motion correction, spatial normalization (MNI), image smoothing, global normalization, and filtering of noise and drift. Echo-planar image acquisition was optimized to preserve signal in ventral prefrontal cortex (see Figure S1). For image analysis, an event-related design modeled the time window bracketing the button responses (16).

### Moral judgment interviews

Two post-scanning interviews were conducted outside the MR scanner, with the results used to determine the personal stage of moral reasoning ability, according to Lawrence Kohlberg's stage model of moral development (1, 1.5, 2,….5). More specifically, each subject's moral ability (development) in the use of an ethic of justice was assessed by using a modified version of Kohlberg's Moral Judgment Interview (mMJI), which consisted of the three justice dilemmas used for the scanner task (Barry dilemma: truth *versus* authority issues; Vendor dilemma: contract *versus* punishment issues; Mary dilemma: law *versus* quality of life issues). In addition, all subjects completed Kohlberg's standardized Moral Judgment Interview (sMJI), which also consists of three justice dilemmas (Heinz dilemma: life *versus* law issues; Judge dilemma: morality and conscience *versus* punishment; Joe dilemma: contract *versus* authority). Both interviews were scored using Colby and Kohlberg's Standard Issue Scoring Manual [61]. The scoring was done by two “blinded” scorers who had previously established their expertise in the use of the MJI scoring system. To increase the fit between the in-scanner reasoning task and the interview reasoning task (face validity), the moral stage used by each subject to support his “chosen issue” or position was used in the calculation of scores. Interrater scoring reliability was estimated to be 0.86 for mMJI stage scores and 0.88 for sMJI stage scores, using the Spearman rho. In addition, moral judgment stage scores on the mMJI and the sMJI were significantly correlated for both ordinal stage scores (Spearman rho = 0.84, p<0001) and continuous weighted scores (Pearson's r = 0.89). These findings support the contention that the scanner-task moral dilemmas were functionally equivalent to the standard interview moral dilemmas [61] and thus reliably estimated the individual stage of moral reasoning ability.

### fMRI data analysis

The fMRI data were analyzed in a two-stage, random effects procedure. In the first stage, the BOLD response for each story segment category for each subject was modeled with the standard canonical hemodynamic response function (cHRF). Parameter estimates of the cHRF were created via within-subject contrasts collapsed across conditions. The resulting summary statistic images were then entered into a second stage analysis that treated each subject as a random variable. In this way, both within- and between-subject variance is accounted for in the model. Prior image analysis [16] was replicated by using Matlab and Statistical Parametric Mapping software (SPM5; Wellcome Department of Cognitive Neurology, http://www.fil.ion.ucl.ac.uk/spm). Signal was adequate in ventral PFC and anterior temporal cortex by inspection. Isolating the neural response to moral issues involved detecting contrasts to neutral story segments. These planned brain-wide, random effects contrast analyses were intended to serve as localization analyses to define care and justice issue processing-related regions of interest (ROI) used to specify the anatomical model representing a parsimonious path model for effective connectivity analysis. For this purpose, an uncorrected threshold (p<0.005, k>5) was used.

The relationship between individual variability in moral ability (development) and the neural response to justice moral issues was assessed in a brain-wide, voxel-wise correlation analysis for the individual mMJI scores and BOLD contrast values for the moral issues. This analysis identified voxel clusters for which the justice issue-related BOLD response was predicted by mMJI score across subjects and was not confined to the four ROI comprising the putative neural processing network for moral cognitions. For this purpose, an uncorrected threshold (p<0.001, k>5) was used.

### Functional connectivity

To initially identify functional brain networks involved in moral cognitions related to care versus justice ethics, PPI analysis was conducted using BOLD time series from seed regions (radius of 5 mm) identified by the neuroactivation analyses (FP: 3, 51, 23 mm), left STS −59, −43, 10 mm; and dorsal posterior cingulate cortex (PCC; 0, −51, 28). This analysis also had localization functions in informing the anatomical model specification for subsequent effective connectivity analysis. A voxel-wise PPI analysis was first conducted at the individual level to identify brain areas exhibiting significantly correlated functional coupling (the slope of regression) with the seed regions during the processing of care or justice moral issues compared with neutral vignettes. For each subject, the activation time course signal in the reference region (*i.e*., the first eigenvariate time series, adjusted by effect of interest) was extracted from the conventional general linear model (GLM) and entered into the PPI analysis as the first regressor representing the physiological variable. A second regressor representing the dilemma/event type (care, justice, neutral) was entered into the PPI analysis as the psychological variable. The PPI between task and activation signal in the reference region was designated as the regressor of interest in the PPI analysis. Group-level paired *t*-tests were conducted. Due to the preliminary and localization goals of this analysis, areas of significant co-activation were identified at an uncorrected threshold of *p*<0.005, k>5 voxels.

### Effective connectivity

Anatomical model specification represented a trade-off between the goal of stipulating the most parsimonious model comprising the fewest ROIs and paths and the need to be sufficiently comprehensive so as to model a neural processing network truly relevant to the moral cognitions of interest. In this attempt we were aided by the consistency of localized neural activations associated with diverse moral stimulus processing tasks, the results of a planned contrast for moral issue versus neutral event for each moral issue type, and the results of a preliminary analysis of functional connectivity using a model-free PPI approach. The rACC (−6, 41, 9 mm) was added as a fourth ROI for the effective connectivity analysis due to its repeated identification as a region of significant task-related functional connectivity with the major ROI, particularly for justice issue sensitivity. What was lacking in developing a parsimonious path model was hodological or functional evidence for specific path directions by which the number of connections could be minimized to make the path model tractable for stable solutions of its fit to the observed data. We therefore tested a path model constrained by the fewest number of structural variables but was unconstrained as to the nature of their interrelationships (paths).

Time series were extracted for each participant for each modeled region. The identified cluster maxima coordinates for the FP, pSTS, PCC, and rACC were used as centroids for spherical volumes of interest (5 mm radius) in effective connectivity analyses. Each ROI included all voxels within a 5-mm radius of the center coordinate to account for variability in groups or conditions. The hemodynamic delay and effects of transitions between blocks were accounted for by shifting the beginning points of task blocks by 6 s and dropping the first two and the last data point from each block. Structural equation models were derived for each condition using a previously described exploratory adaptation of this modeling method [67]. This adaptation is a brute-force approach that tests every possible model that could be generated from a given dataset, and then ranks the models' goodness-of-fit criteria to find the model with the least discrepancy between the predicted and observed variance-covariance matrices. Exploratory SEM was implemented in Matlab R2007a (The MathWorks, Inc.) and Lisrel 8.80 (Scientific Software International). A separate analysis sought to compare path strengths or weights between the three issue conditions for the stipulated path model. T-tests compared the β values for each connection with a Bonferroni correction based on adjustment of α by the observation that a total of 19 paths were shared by two or more conditions. This analysis was limited by the ability to compare only those paths in the model for which SEM derived significant connectivity coefficients.

In addition, a stepwise multiple regression analysis was performed to determine which (if any) of the participants' SEM path coefficients for the justice model (generated during justice issue sensitivity) could predict their mMJI scores. This analysis was performed by first fitting each participant to the group-derived justice model. In other words, our exploratory adaptation of SEM found the model that best fit the entire group, then “regular” confirmatory SEM assessed how well each subject fit the group model. A multiple regression that used individual participants' path coefficients to predict participants' mMJI scores was performed in SPSS 17.0 (SPSS, Inc.). The regression used mMJI scores as the dependent variable and each SEM path coefficient as input variables. The stepwise procedure was invoked with an inclusion threshold of p<0.05 and exclusion threshold of p>0.10. That is, the path coefficient with the greatest partial correlation with mMJI scores was added to the regression equation if its contribution to r^2^ was significant (F-test, p<0.05), and a path coefficient was removed from the regression equation if its partial correlation with mMJI scores was no longer significant after inclusion of another variable (F-test, p>0.10).

## Supporting Information

Figure S1 A representative EPI image depicts preservation of signal in ventral frontal and temporal lobes.
(5.12 MB TIF)

Table S1 Influence of individual variation in moral ability (ethics of justice) on neural responses related to moral sensitivity for justice issues (p<0.001, k> = 5).
(0.05 MB DOC)
