# Supplementary figures and images for: Mode of Effective Connectivity within a Putative Neural Network Differentiates Moral Cognitions Related to Care and Justice Ethics
Source: PLoS One. 2011 Feb 25;6(2):e14730. doi: 10.1371/journal.pone.0014730 (PMC3045376; doi:10.1371/journal.pone.0014730)

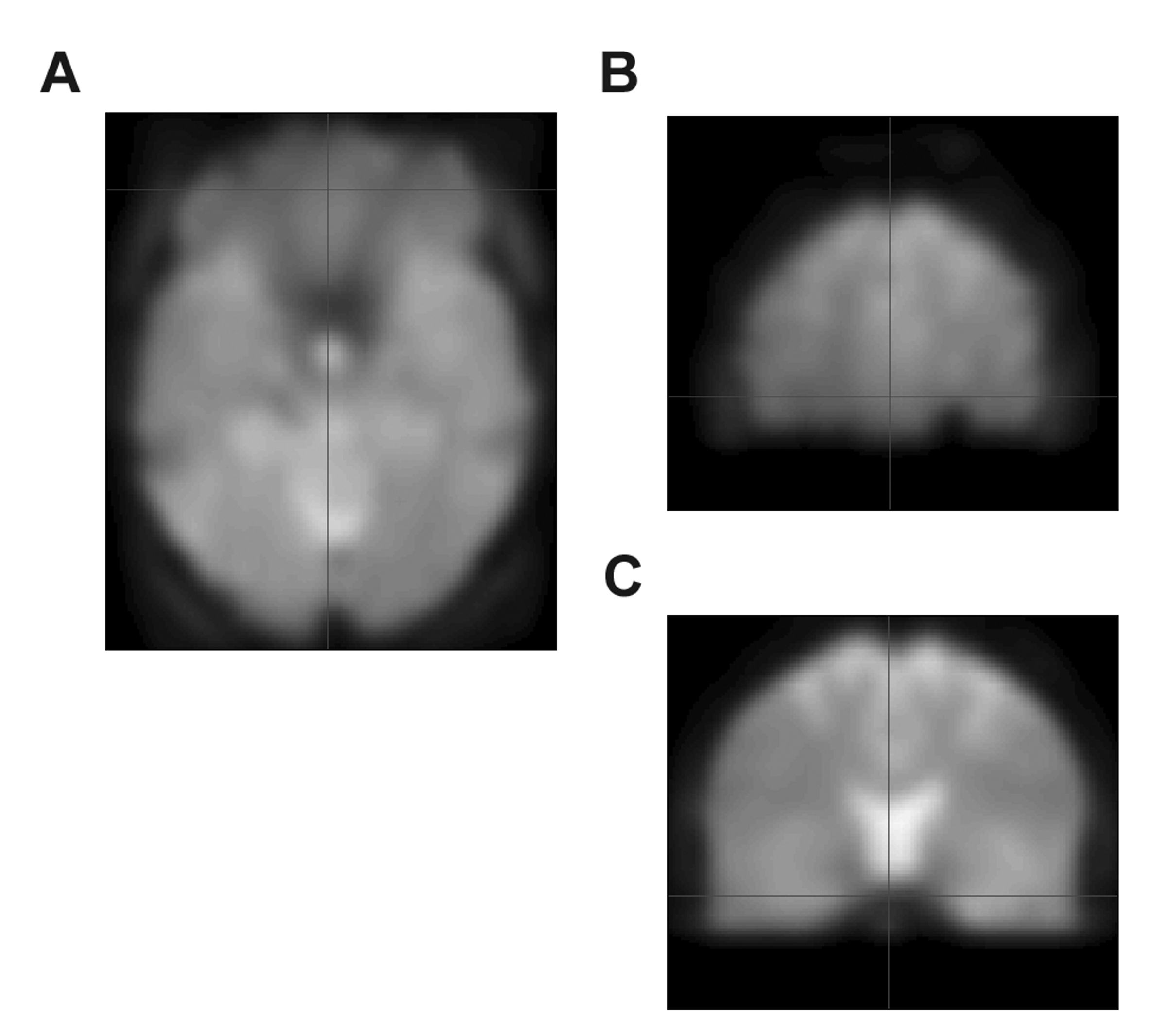

Supplement: Figure S1 — A representative EPI image depicts preservation of signal in ventral frontal and temporal lobes. (5.12 MB TIF) [file pone.0014730.s001.tif]
