# Supplementary material for: Mode of Effective Connectivity within a Putative Neural Network Differentiates Moral Cognitions Related to Care and Justice Ethics
Source: PLoS One. 2011 Feb 25;6(2):e14730. doi: 10.1371/journal.pone.0014730 (PMC3045376; doi:10.1371/journal.pone.0014730)
Supplement: Supplementary file 2 [file pone.0014730.s002.doc]

Table S1. Influence of individual variation in moral ability (ethics of justice) on neural responses related to moral sensitivity for justice issues ($p < 0.001$, $k \geq 5$).

Contrast	Region	Voxel T	Cluster size	Tailarach coordinates x	y	Z
Justice > Neutral: correlation with mMJI scores						
Positive						
	SMA (6)	5.86	51	0	-14	71
	STS (L 22)	4.89	41	-53	-37	13
Negative						
	Dorsal ACC (R 32)	5.6	72	21	11	36
Justice > Neutral: median split analysis						
High > low						
	STS (L 22)	4.92	19	-56	-37	13
	Pre-SMA (6)	4.37	5	-6	15	66
	SMA (6)	4.35	13	-3	-8	70
Low > high						
	Dorsal ACC (L 24)	6.13	10	-18	2	37
	Cuneus (19)	5.02	5	9	-80	33
	Dorsal ACC (R 32)	4.95	18	18	11	36
	Middle temporal gyrus (L 21)	3.58	5	-48	-15	-3
	Superior frontal gyrus (R 10)	3.5	5	24	64	-3
	Precentral gyrus (R 4)	3.26	7	36	-6	48

Letters/Numbers in parentheses refer to Hemisphere/Brodmann Area

Abbreviations: SMA, supplementary motor area
